# Is Lipoprotein-Associated Phospholipase A2 a Link between Inflammation and Subclinical Atherosclerosis in Rheumatoid Arthritis?

**DOI:** 10.1155/2015/673018

**Published:** 2015-10-04

**Authors:** Anna Södergren, Kjell Karp, Christine Bengtsson, Bozena Möller, Solbritt Rantapää-Dahlqvist, Solveig Wållberg-Jonsson

**Affiliations:** ^1^Department of Public Health and Clinical Medicine/Rheumatology, University of Umeå, 901 87 Umeå, Sweden; ^2^Department of Surgical and Perioperative Sciences, University of Umeå, 901 87 Umeå, Sweden; ^3^Department of Rheumatology, Östersund Hospital, 831 27 Östersund, Sweden; ^4^Department of Rheumatology, Sunderby Hospital, 971 89 Luleå, Sweden

## Abstract

*Objective*. Lipoprotein-associated phospholipase A2 (Lp-PLA2), a marker of vascular inflammation, is associated with cardiovascular disease. This prospective study of an inception cohort aimed to investigate whether the level of Lp-PLA2 is associated with subclinical atherosclerosis in patients with rheumatoid arthritis (RA). *Methods*. Patients from northern Sweden diagnosed with early RA were consecutively recruited into an ongoing prospective study. From these, all patients ≤60 years (*n* = 71) were included for measurements of subclinical atherosclerosis at inclusion (T0) and five years later (T5). Forty age- and sex-matched controls were included. The patients were clinically assessed, SCORE, Reynolds Risk Score, and Larsen score were calculated, and blood samples were drawn from all individuals at T0 and T5. *Results*. There was no significant difference in the level of Lp-PLA2 between patients with RA and controls (*p* > 0.05). In simple linear regression models among patients with RA, Lp-PLA2 at T0 was significantly associated with intima media thickness (IMT) at T0 and T5, flow mediated dilation (FMD) at T0 and T5, ever smoking, male sex, HDL-cholesterol (inversely), non-HDL-cholesterol, SCORE, Reynolds Risk Score, and Larsen score (*p* < 0.05). *Conclusion*. In this cohort of patients with early RA, the concentration of Lp-PLA2 was associated with both subclinical atherosclerosis and disease severity.

## 1. Introduction

Patients with rheumatoid arthritis (RA) have increased atherosclerosis compared with the general population [1–4]. Atherosclerosis is now recognised as an inflammatory disease* per se *[5] and the two diseases, atherosclerosis and RA, are considered to share many similarities [6], albeit the link between them is, as yet, not evident.

Subclinical atherosclerosis precedes cardiovascular disease (CVD) and an increased intima media thickness (IMT), measured by ultrasonography, is regarded as an early indicator of a generalized atherosclerosis [7]. Several studies in the general population, as well as in patients with RA, have shown a relationship between an increased IMT and a future cardiovascular event [8–12]. We, and others, have previously shown that patients with established RA have a premature atherosclerosis as measured by an increased IMT of the common carotid artery (CCA) compared with controls [13, 14]. An even earlier sign of atherosclerosis, that is, endothelial dysfunction, indicated by an impaired flow mediated vascular dilation (FMD) of peripheral arteries, can also be measured using ultrasonography [15]. In the general population, FMD has been associated with other established risk factors for CVD and shown to be predictive of a future CV event [15–17].

Lipoprotein-associated phospholipase A2 (Lp-PLA2), formerly also known as platelet-activating factor acetylhydrolase (PAF-AH), is an enzyme expressed, among others, by inflammatory cells in atherosclerotic plaques [18, 19]. Lp-PLA2 hydrolyses phospholipids in low-density lipoprotein (LDL) to yield proinflammatory products such as oxidized free fatty acids. These proinflammatory products play a critical role in the endothelial chemotactic response by stimulating the expression of adhesion molecules and cytokines as well as recruiting inflammatory cells. Hence, Lp-PLA2 is suggested to be a useful and potent biomarker of the vascular inflammation involved in the pathophysiology of atherosclerosis [19–21].

In the circulation, Lp-PLA2 is carried bound mainly to LDL, and several epidemiological studies in the general population have shown a correlation between Lp-PLA2 levels and traditional cardiovascular risk factors [18, 22, 23]. In the general population, higher concentrations of Lp-PLA2 have also been shown to be associated with an increased risk of CVD [18]. Previous studies of patients with thalassemia, metabolic syndrome, or human immunodeficiency virus (HIV) infection, each diagnosis being characterised by an increased inflammation, have shown an association between Lp-PLA2 and subclinical atherosclerosis measured by IMT [20, 23, 24]. In populations without a known inflammation, however, the results are contradictory [21, 25–27]. To the best of our knowledge there are no studies on the relationship between Lp-PLA2 and atherosclerosis in patients with RA.

In an ongoing prospective case-control study of patients with very early RA [28], we have found a significant increase in the subclinical atherosclerosis, measured by IMT and FMD, during the first five years of rheumatic disease [29]. In the present study, we hypothesized that vascular inflammation, reflected by the concentration of Lp-PLA2, contributes to the atherosclerotic disease in patients with RA. Thus, our primary aim was to investigate whether the level of Lp-PLA2 was associated with subclinical atherosclerosis at baseline (T0) or after the first five years following a diagnosis of RA (T5). A secondary aim was to identify markers of inflammation and traditional CVD risk factors associated with Lp-PLA2 and hence with a possible vascular inflammation preceding CVD.

## 2. Material and Methods

### 2.1. Patients and Controls

The present study is a part of a continuing structured programme on early RA for prospective analysis of CVD development in patients from northern Sweden using the nationwide Swedish Rheumatoid Arthritis Registry. All eligible patients with newly diagnosed RA (according to ACR criteria) [30] and symptomatic for no longer than 12 months are continuously enrolled into the register as soon as possible following diagnosis (T0). The inclusion criteria for the patients with RA and controls have previously been described in detail [28]. Five years after inclusion into the study (T5), 71 of the 79 patients with RA originally included were willing to participate in the follow-up study, and 40 of the original 44 controls were reassessed. The controls (one control for two patients except for in 13 cases one control per patient) were matched for age (**±**5 years) and sex. Only those individuals participating in the follow-up assessment, that is, T5, were included in this study. All individuals gave their written consent in accordance with the Declaration of Helsinki. The study was approved by the Regional Ethics Committee of Umeå University, Umeå, Sweden.

### 2.2. Physical Examination and Surveys

All patients were examined clinically at inclusion into the study and regularly thereafter at 3, 6, 12, 18, 24, and 60 months. The number of swollen and tender joints (28-joint count) and the patient's global assessment were registered, and a disease activity score (DAS28), including the erythrocyte sedimentation rate (ESR), was calculated [31]. Posterior-anterior radiographs of the hands, wrists, and feet were obtained at baseline and after five years and were graded according to the Larsen score by two rheumatologists (EB and Solbritt Rantapää-Dahlqvist) [32]. All participants completed a survey on comorbidity. Any previous CVD events were verified by reference to medical records. Up to the five-year follow-up assessment, eight (11%) of the patients with RA had suffered a CVD event (3 acute myocardial infarction, 3 stroke, and 2 thromboembolic event) whilst two (5%) of the controls had suffered a CVD event (both coronary artery bypass graft surgery). Blood pressure was measured at the time of ultrasound measurements. Body mass index (BMI), European Systematic Coronary Risk Evaluation (SCORE) [33], and Reynolds Risk Score [34] were calculated at both T0 and T5. These compound measures of CVD risk factors estimate the risk of death due to a CVD event during the next 10 years. In addition to traditional CVD risk factors, the Reynolds Risk Score includes C-reactive protein (CRP) concentrations. When calculating the Reynolds Risk Score, all patients were regarded as being nondiabetic due to a lack of information regarding levels of haemoglobin A1c for all individuals; this assumption may have resulted in an underestimation of the risk score.

### 2.3. Ultrasound Investigations

The patients were examined by ultrasound as soon as possible following diagnosis (at T0); the mean (±SD) time after the primary symptom of RA was 16.2 (±6.6) months. The ultrasound investigations at the follow-up (T5) were performed 5 years after the initial examinations (mean and median being 60 months, with a range 59–63, after the first examination). All ultrasonography examinations of patients with RA and controls were performed by the same experienced investigator (EL); the individuals were in a supine position in a quiet, temperature controlled room. A Sequoia 512 ultrasound system (Siemens (Acuson) Corp) was used with a 15L8 transducer for measurement of the brachial artery and an 8L5 transducer for carotid artery studies. All investigations were digitally stored for analyses to be performed by the single observer (EL; intraobserver variability for IMT *r* = 0.988). The protocol for these investigations has previously been described in detail [28].

### 2.4. Blood Sampling

In the present study all patients and controls donated a blood sample at the time of both ultrasound measurements, that is, at T0 and T5, and serum was stored at −80°C. After thawing, serum concentrations of Lp-PLA2 (ng/mL) were measured using an ELISA (R&D Systems, Abingdon, UK). Rheumatoid factor (RF; 67% of the patients were seropositive), CRP (mg/L), and erythrocyte sedimentation rate (ESR; mm/h) were measured according to routine methodology. Whenever several analyses of DAS28, CRP, or ESR were performed on any given individual, the assessment closest to the ultrasound measurement was used in any subsequent statistical analysis. Blood was also drawn after an overnight fast for analysis of blood lipids: cholesterol (mmol/L), high-density lipoproteins (HDL; mmol/L), and triglycerides (mmol/L) using routine methods at each of the participating hospitals. Lp-PLA2 concentration results were available for 70 and 66 patients with RA at T0 and T5, respectively. Correspondingly, results for Lp-PLA2 were available for 38 and 40 controls at T0 and T5, respectively.

### 2.5. Statistics

Comparisons over time within the RA patient group and within the control group were performed using the Wilcoxon paired test. Simple and multiple linear regression analyses were used to identify variables associated with Lp-PLA2. Results from simple linear regression (variables with *p* < 0.05), together with clinical assumptions (variables with *p* > 0.05), determined which covariates were included in the multiple linear regression models. Differences in variables between patients with RA and matched controls were analysed using simple conditional logistic regression analyses. Occasional missing values, due to missing information, were regarded as random. Non-HDL-cholesterol was calculated as total cholesterol minus HDL-cholesterol. Based on previously published data [20], calculations showed that a sample size of 71 would render 99% power to detect a correlation between IMT and Lp-PLA2 with a correlation coefficient of 0.46. *p* values < 0.05 were considered statistically significant. All calculations were made using SPSS 20.0 (SPSS Inc., Chicago, USA).

## 3. Results

For this study, 71 patients with RA (61 (86%) women) and 40 controls (32 (80%) women) were included. The mean age (SD) of the patients with RA was 51.5 (10.7) years and 48.1 (10.9) years for the controls. Among the patients with RA, 35 (58%) declared themselves to ever being a smoker, corresponding with 14 (39%) among the controls. Six (9%) patients with RA and 5 (12%) controls had ever used statins.

The concentration of Lp-PLA2 increased significantly during the 5-year follow-up period, both for the patients with RA and the controls (Table 1). At both time points the concentrations of Lp-PLA2 were numerically higher in patients with RA compared with controls (*p* values >0.05) (Table 1).

Among the patients with RA, the concentration of Lp-PLA2 at T0 was significantly associated with IMT as well as with FMD at both baseline and follow-up (Table 2). After adjustment for sex and age, Lp-PLA2 was still significantly associated with IMT at T0 and T5 (Table 2). At T0, the Lp-PLA2 concentration was also significantly associated with non-HDL-cholesterol, HDL (inversely), diastolic blood pressure, smoking, SCORE, and Reynolds Risk Score as well as the Larsen score (Table 2). Adjustment for disease activity, measured by DAS28 at T0, did not change significantly the association between the Lp-PLA2 levels at T0 and disease severity measured by the Larsen score at T0 (Table 3).

At T5 Lp-PLA2 was significantly associated with non-HDL-cholesterol, HDL (inversely), cholesterol, BMI, and Reynolds Risk Score among the patients with RA (Table 4). There were no associations between Lp-PLA2 and any of the measures of disease activity, that is, CRP, ESR, and DAS28, at either T0 or T5 (data not shown). Furthermore, there was no significant association between Lp-PLA2 concentration and the Larsen score at T5, nor were there any significant associations between Lp-PLA2 and age, or any medication either at T0 or at T5 (data not shown).

Among the controls, Lp-PLA2 at T0 was significantly associated with IMT at T0 (*β* 3.2, *p* = 0.05) and Lp-PLA2 at T5 was significantly associated with several variables measured at T0: IMT (*β* 3.9, *p* < 0.05), cholesterol (*β* 6.1, *p* < 0.001), triglycerides (*β* 3.3, *p* < 0.05), diastolic blood pressure (*β* 4.1, *p* = 0.01), age (*β* 5.2, *p* < 0.001), and SCORE (*β* 4.1, *p* < 0.05). Among the same individuals Lp-PLA2 at T5 was significantly associated with IMT (*β* 3.3, *p* < 0.05) and cholesterol (*β* 5.8, *p* < 0.001) at T5. After adjustment for sex and age no significant association between Lp-PLA2 and IMT was found among the controls, neither at T0 nor at T5 (data not shown).

## 4. Discussion

In this study, the serum concentration of Lp-PLA2 was associated with measures both of subclinical atherosclerosis over time and of disease severity at disease onset in patients with early RA.

From previously published reports, it is evident that patients with RA have an increased development of atherosclerosis compared with the general population, with different underlying causes being proposed to explain this observation [3, 4, 6, 13]. A strong theory to date is that the inflammatory load among the patients with RA affects the arteries and gives rise to a subclinical vascular inflammation. An increased level of Lp-PLA2 among patients with RA was shown several years ago, when it was presented as a marker of disease activity among such patients [35, 36]. However, more recently published studies on other inflammatory diseases, as well as on the general population, suggest that Lp-PLA2 cannot be regarded as a marker of a systemic inflammation but as a mere biomarker of atherosclerosis [19]. With this background in mind, we measured the concentration of Lp-PLA2 as a marker of vascular inflammation. However no significant difference in the levels of Lp-PLA2 in patients with RA and controls was found, neither early in the disease nor after 5 years, albeit the concentrations of Lp-PAL2 were numerically higher among the patients with RA at all time points.

It is now recognized that atherosclerosis is the result of an inflammatory process in the vessel wall [5], and early atherosclerosis can be identified as an endothelial dysfunction by FMD or arterial wall thickening by IMT. The Lp-PLA2 concentration at inclusion of patients with early RA into this study, as well as at the five-year follow-up assessment, was found to be associated with both measurements of early atherosclerosis. Lp-PLA2 is regarded as a highly specific biomarker for vascular inflammation and burden of atherosclerosis [19]. The correlation between this biomarker and atherosclerosis is well studied in the general population, as well as in other inflammatory diseases; however there are also contradictory results [20, 21, 23–27]. After five years following a diagnosis of RA, the levels of Lp-PLA2 found at inclusion could still explain the extent of atherosclerosis measured by IMT and FMD in patients with RA. There are, to the best of our knowledge, only a few prospective studies on Lp-PLA2 and the development of subclinical atherosclerosis and none regarding patients with an inflammatory disease. Two studies on patients with diabetes mellitus found measurements of Lp-PLA2 to be associated with the progression of atherosclerosis over time [37, 38] and Liu et al. verified these results in the general population [27]. In this study a similar result was found, with an association between Lp-PLA2 and prospectively registered measures of atherosclerosis in patients with early RA.

In previous studies involving this cohort of patients, the extent of atherosclerosis was associated with traditional cardiovascular risk factors [28, 29]. In the present study we found, consistent with other published studies, the levels of Lp-PLA2 to be associated with several traditional risk factors [18, 22, 23]. Moreover, the compound measure of CVD risk that included inflammation, that is, the Reynolds Risk Score, was strongly associated with Lp-PLA2 at both T0 and T5. The Lp-PLA2 molecule is carried in the circulation mainly bound to LDL [18, 19] and, as was to be expected, the level of Lp-PLA2 was found to be evidently associated with the concentrations of blood lipids. Non-HDL-cholesterol, in some regards a better measurement of the risk of CVD than LDL [39], was strongly associated with Lp-PLA2 both at T0 and at T5.

Radiological progression is a measurement of disease severity over time in patients with RA. We found a significant relationship between Lp-PLA2 concentration and the Larsen score at the time of diagnosis of RA. In these patients, the same inflammatory process that leads to joint damage may also affect the vascular walls causing a vascular inflammation, as reflected by elevated concentrations of Lp-PLA2. This association was not altered significantly by adjustment for disease activity at inclusion, again indicating that Lp-PLA2 is not just a marker of disease activity. In a multiple regression model, both IMT and Larsen score at inclusion were significantly associated with the concentration of Lp-PLA2, indicating that the processes leading to joint damage and vascular damage are, in some part, interlinked.

The main strength of the present study is the prospective design from the onset of disease. In northern Sweden almost all individuals newly diagnosed with RA are included in a structured follow-up programme. Of these patients, all of those aged ≤60 years were invited to participate in this study within 12 months of their diagnosis. Data on biomarkers and traditional CVD risk factors, as well as variables related to the RA disease, were collected from the onset of disease and then continuously during the five years of follow-up. Another strength of this study is that the same person (KE) undertook all of the laboratory-based analyses and that Lp-PLA2 in samples collected at both time points (i.e., T0 and T5) was measured simultaneously. Furthermore, all ultrasound measurements at both time points, and their analysis, were undertaken by the same person (EL).

Conversely, a limitation of this study is that it is strictly observational; in other words, no influence could be made on medications and other variables observed. Another limitation is the number of control subjects; however this study was directed primarily at studying the serum concentrations of Lp-PLA2 among the patients with RA. Another limitation is the lack of data on LDL-cholesterol levels. However, non-HDL-cholesterol was calculated, based on findings reported in some studies that it is more strongly associated with a risk of CVD than LDL-cholesterol [39]. The relationship between Lp-PLA2 and LDL-cholesterol must be regarded as well studied [18, 22]. Furthermore, we were not able to explore the association between Lp-PLA2 and CVD since there were too few CV events during the follow-up. Still, this study is the first of its kind, and further studies, including a follow-up of the individuals in the present study, will be able to clarify this association.

## 5. Conclusions

In this study, the level of Lp-PLA2 among patients with RA was associated with subclinical atherosclerosis, prospectively measured by IMT and FMD. Among these patients with early RA, this biomarker of vascular inflammation was also associated with Larsen score, indicating that over time the deleterious disease process may also affect the vascular walls. Taken together, our findings indicate a continuous vascular inflammation among patients with RA possibly leading to the development of atherosclerosis and hence to CVD. This possibility adds to the knowledge of the mechanisms responsible for the observed increased risk of CVD among patients with RA.

## Figures and Tables

**Table 1:** Measurement of Lp-PLA2, intima media thickness (IMT), flow mediated dilatation (FMD), traditional risk factors for cardiovascular disease (CVD), and disease activity in patients with early rheumatoid arthritis (RA) and in age- and sex-matched controls evaluated both at baseline (T0) and after 5 years (T5). Data are expressed as mean value (standard deviation).

	RA (*n* = 71)		Controls (*n* = 40)	
	At T0	At T5	At T0	At T5
Lp-PLA2, ng/mL	144.0 (41.7)	154.6 (38.9)^∗^	132.0 (37.8)	148.1 (41.7)^∗∗^
Intima media thickness, mm	0.52 (0.13)	0.58 (0.13)^∗∗∗^	0.54 (0.13)	0.60 (0.12)^∗∗∗^
Endothelium dependent flow mediated vasodilatation, %	109.2 (4.7)	107.0 (4.7)^∗∗∗^	107.2 (4.5)	106.0 (4.6)
Systolic blood pressure, mmHg	123.5 (14.4)	126.3 (13.9)^∗^	117.7 (11.3)	124.1 (12.1)^∗∗∗^
Cholesterol, mmol/L	5.5 (0.9)	5.3 (1.0)	5.3 (1.1)	5.6 (1.1)
HDL, mmol/L	1.6 (0.5)	1.6 (0.5)	1.5 (0.4)	1.7 (0.5)
Non-HDL-cholesterol, mmol/L	3.9 (0.9)	3.7 (1.0)	3.9 (1.1)	3.9 (1.1)
Triglycerides, mmol/L	1.3 (0.5)	1.2 (0.5)	1.1 (0.3)	1.0 (0.5)
BMI, kg/m^2^	25.8 (4.0)	25.7 (4.5)	25.1 (4.9)	25.2 (4.2)
CRP, mg/L	11.9 (10.8)	7.7 (7.2)^∗∗^	n/a	n/a
DAS28	3.5 (1.4)	3.1 (1.5)^†^	n/a	n/a

^∗^ *p* value < 0.05; ^∗∗^ *p* value < 0.01; ^∗∗∗^ *p* value < 0.0001; ^†^ *p* value = 0.061; all values compared with T0.

Lp-PLA2: lipoprotein-associated phospholipase A2; HDL: high-density lipoproteins; BMI: body mass index; CRP: C-reactive protein; DAS28: disease activity score for 28-joint count.

**Table 2:** Results of simple regression models among the 71 patients with early RA with the concentration of Lp-PLA2 at T0 as the dependent variable.

	Lp-PLA2 at T0		
	*β*	95% CI	*p* value
IMT T0 (*n* = 70)	9.7/mm	2.1; 17.2	0.013^†^
IMT T5 (*n* = 70)	8.8/mm	1.4; 16.2	0.02^†^
FMD T0 (*n* = 70)	−2.4/%	−4.5; −0.4	0.02
FMD T5 (*n* = 70)	−2.5/%	−4.6; −0.5	0.02
Non-HDL-cholesterol T0 (*n* = 54)	16.9/mmolL^−1^	5.8; 28.0	0.004
HDL T0 (*n* = 55)	−22.6/mmolL^−1^	−42.0; −3.1	0.02
Diastolic blood pressure T0 (*n* = 66)	1.3/mmHg	0.1; 2.5	0.04
Ever smoking (*n* = 70)	0.8/year	0.08; 1.4	0.03
SCORE T0 (*n* = 53)	11.4/unit	3.3; 19.5	0.007
Reynolds Risk Score T0 (*n* = 38)	5.2/unit	1.5; 8.8	0.007
Larsen score T0 (*n* = 50)	2.9/unit	0.013; 5.90	0.05

^†^Still significant after adjustment for sex and age.

RA: rheumatoid arthritis; Lp-PLA2: lipoprotein-associated phospholipase A2; IMT: intima media thickness; FMD: flow mediated dilation; HDL: high-density lipoproteins.

**Table 3:** Multiple regression models among 71 patients with early RA with the concentration of Lp-PLA2 at T0 as dependent variable.

	Lp-PLA2 at T0		
	*β*	95% CI	*p* value
Larsen score T0	2.8/unit	−0.3; 5.8	0.06
DAS28 T0	−4.6/unit	−2.0; 0.3	0.03

RA: rheumatoid arthritis; Lp-PLA2: lipoprotein-associated phospholipase A2; DAS28: disease activity score for 28-joint count.

**Table 4:** Results of simple regression models among 71 patients with early RA with the concentration of Lp-PLA2 at T5 as the dependent variable.

	Lp-PLA2 at T5		
	*β*	95% CI	*p* value
IMT T5 (*n* = 66)	4.4/mm	−2.7; 11.5	0.22
FMD T5 (*n* = 66)	−1.7/%	−3.8; 0.4	0.10
Non-HDL-cholesterol T5 (*n* = 61)	19.0/mmolL^−1^	10.5; 27.5	0.001
HDL T5 (*n* = 61)	−31.8/mmolL^−1^	−50.3; −13.3	0.001
Cholesterol T5 (*n* = 61)	11.5/mmolL^−1^	2.1; 20.8	0.02
Ever smoking (*n* = 66)	0.4/year	−0.3; 1.1	0.26
BMI T5 (*n* = 66)	2.5/unit	0.3; 4.7	0.03
SCORE T5 (*n* = 61)	3.6/unit	−2.0; 9.2	0.21
Reynolds Risk Score T5 (*n* = 39)	2.8/unit	−0.2; 5.7	0.06

RA: rheumatoid arthritis; Lp-PLA2: lipoprotein-associated phospholipase A2; IMT: intima media thickness; FMD: flow mediated dilation; HDL: high-density lipoproteins; BMI: body mass index.
